# The Effect of Variations in Food on the Digestive Glands

**Published:** 1903-02-28

**Authors:** 


### Hospital Clinics and Medical Progress.

### THE EFFECT OF VARIATIONS IN FOOD ON THE DIGESTIVE GLANDS.

A special adaptation of secretion to food is seen in all the digestive juices. This adaptation is seen in the salivary glands, though they differ in some other respects from the abdominal digestive glands. The saliva has a variety of functions to perform. Not only have widely different foods to be masticated and prepared for swallowing, but noxious substances have to be removed from the mouth and all traces washed away.

Hence it was found by Pawlow and his pupils that a copious watery secretion from the salivary glands was evoked by pouring dry sand into the mouth, or irritating the buccal mucous membrane with acids. The acids were particularly active in stimulating the parotid secretion of the dog. These observations account for the secretion of saliva aroused by feelings of disgust, and the secretion of saliva that frequently precedes the act of vomiting.

But the adaptation is best seen by comparing the effect of stimulating the glands with different substances. Edible substances, notably raw flesh, called forth a thick mucoid secretion, capable of welding the food into a bolus. This secretion does not come from the parotid gland of the dog, but from the submaxillary and sub-lingual glands. If now the animal be fed with powdered dry flesh, there is a copious parotid secretion in addition to that of the other salivary glands; and this though the animal is much more excited and eager when fed with the raw meat. Similarly, the parotid secretion is evoked by feeding with dry bread, but not with moist bread. Here, then, is a definite example of the purposive nature of the secretion.

The nature of the food ingested has a definite relation with the properties of the digestive secretion evoked. Each variety of food, proteid, carbohydrate, and fat, calls forth a particular kind of secretion. Flesh evokes a gastric secretion that reaches its maximum rate in about one and a half or two hours. The digestive power of this secretion is greatest at the end of the first hour, it then falls, but rises again at the end of five or six hours. In the case of bread, the total secretion is small, the maximum rate of secretion is reached in one hour and then rapidly falls off. The strength of the secretion, as measured by its digestive power, increases until the end of the second hour and then diminishes. Milk, however, evokes a totally different response. The rate of secretion is small at first, but reaches a maximum in two hours, and this is maintained through the third hour after the ingestion of food. Also the strength of the secretion, fairly high at first, falls during the first hour, but afterwards gradually rises again.

These experimental facts show that both the character and nature of gastric secretion are directly dependent upon the nature of the food. Analysis of the facts points out the extent of the variations. The rapid and effective juice poured out on flesh, compared with the scanty secretion in the case of bread, or the delayed secretion in the case of milk. Why this should be so has been explained by further experiments by the pupils of Pawlow. The secretion evoked by bread is that caused by proteid and starch in the amounts present in bread. This was proved by making a starch jelly containing proteid in the amount normally present in bread, and this caused a similar secretion of gastric juice.

The delayed secretion in the case of milk was shown to be due to the fat contained therein. Equal quantities of milk and cream, introduced into the stomach, caused only half the secretion in the case of the cream compared with that evoked by the milk. In another experiment, the previous introduction of 75 c.cm. of Provence oil reduced the gastric secretion evoked by a flesh meal down to one quarter of the normal amount. Fat, then, inhibits the gastric secretion. This explains the "indigestion" often noticed after eating fatty foods containing some proteid. The digestion of the proteid is impeded by the inhibitory action of the fat.

Fat alone is not particularly "indigestible," passing direct into the duodenum, where it evokes a pancreatic secretion peculiarly suitable for its proper digestion. Bread and butter is also more readily dealt with than fat and proteid. The bread requires but a minimum of gastric secretion, and the butter evokes a strong pancreatic secretion also necessary to deal with the carbohydrates of the bread.

Returning to the secretion evoked by the ingestion of milk, it is found to be the weakest gastric juice of all, and in addition the pancreatic juice secreted is the smallest in amount. That is when an equivalent quantity of nitrogenous food is given, as flesh, bread, or milk, the smallest secretory activity is evoked in the case of milk. Milk has further the important property of being an independent exciter of the digestive glands. Even when brought into the stomach unnoticed there is an early secretion of gastric juice. But the total functional activity, evoked by this equivalent amount of milk, is only one quarter of that aroused by a bread diet of the same value. The secretion poured out on the milk is effective, but at the same time economic.

The importance of milk as a food from this is apparent, particularly when economy of digestive-gland-activity is of moment. The utilisation of food-stuffs depends on more than the mere fraction of food digested, in its passage through the alimentary canal of the animal. In vitro, fibrin is readily and completely digested, but not so milk. In the dog, compare the value of fibrin and milk. The former is rapidly digested and absorbed, much more so than the latter; but the fibrin stimulates the digestive glands to a far greater degree of secretory activity. Here is the important point in the value of a food, namely the corresponding secretory activity of the digestive glands. In the case of milk this is small, and herein lies the suitability of milk as a food.

The nature of the food ingested also influences the amount of bile secreted, and with many foods the parallelism of biliary and pancreatic secretion is most marked. The flow of bile lasts throughout digestion, but the amount depends on the nature of the food. Fat and the extractives of meat promote the flow of bile, while pure proteids, *e.g.* white of egg, do not. Bile possesses a digestive importance, in that it neutralises the acid chyme from the stomach, and so provides a favourable medium for the action of the pancreatic ferments.

The pancreatic secretion may, however, contain very small quantities of active ferment. In this condition it usually contains in addition large quantities of the precursor of the ferment. To activate this precursor, or zymogen, the succus entericus is necessary. The succus entericus contains an activating body or ferment termed "enterokinase," which, when added to the pancreatic zymogen is a most powerful proteolytic ferment. This is only another of the many links in the complex alliance of the digestive glands.

